# Editorial: The Cell Biology of Protist Parasite-Host Interfaces

**DOI:** 10.3389/fcell.2022.866421

**Published:** 2022-02-22

**Authors:** Carmen Faso, Adrian B. Hehl

**Affiliations:** ^1^ Institute of Cell Biology and Multidisciplinary Center for Infectious Diseases, University of Bern, Bern, Switzerland; ^2^ Institute of Parasitology, University of Zürich, Zürich, Switzerland

**Keywords:** parasite-host interactions, protist, cell-invasion, protein-trafficking, immune-evasion strategies, parasite nutrition

Molecular exchange at the parasite-host interface is key to parasite survival since these eukaryotic species feed off of host-acquired nutrients and often complete key stages of their life-cycles within them. The focus of this special issue is on our level of mechanistic understanding of the complex communication established between a parasite and a host, with a focus on protist parasites which cause significant morbidity and disability worldwide in both medical and veterinary contexts. Protist parasites have evolved an incredibly diverse range of strategies to invade their hosts and ensure survival and the cell biology underlying interactions and protein/metabolite trafficking at protist parasite-host interfaces, whether extra- or intra- cellular, is a field of intense investigation.

In this special issue, contributions from experts in Plasmodium, Toxoplasma, Theileria, Giardia, Leishmania, Trypanosoma and Entamoeba cell biology provide critical reviews and experimental data on essential topics such as parasite nutrition, parasite surface remodeling, protein trafficking and parasite biology in terms of invasion and niche establishment.

Focusing on parasite cell biology, Zeeshan et al. report on the essentiality of parasite kinesin 5 for the production of infectious *Plasmodium berghei* sporozoites. They are joined by Thomas et al. in their investigation of the role for *Gl*Rac in defining progression and maturation of organelles essential for infectious cyst formation in *Giardia lamblia*. A more “omics”-based approach enables Sun et al. to investigate global proteomics changes in both host and parasite cells during *Toxoplasma gondii* infection while König et al. compare and contrast the predicted proteome of *Entamoeba histolytica* and its sister species to discern lineage-specific genes which may help explain virulence patterns in otherwise closely-related species. With a focus on Leishmania pathogenesis, Salloum et al. discuss insight from RNA-Seq based analyses and present an exciting outlook for investigation which includes the host’s/vector’s microbiota in the equation for a parasite’s pathobiology.

Critical reviews on parasite-dependent hijacking of host cell-specific pathways include an in-depth discussion and appreciation for the role of autophagy in *Toxoplasma gondii* infection and establishment in nervous tissue by Subauste, joined by Woods et al. for a discussion of strategies Theileria parasites to invade and immortalize leukocytes. For a stronger focus on parasite nutrition, Counihan et al. discuss recent advances in our understanding of how Plasmodium parasites take up nutrients from the host cell milieu, including the role for endocytosis in ferrying hemoglobin to the parasitophorous vacuole. Endocytosis and protein trafficking routes involved in parasite virulence are discussed in three manuscripts. Link et al. discuss secretory and endosomal pathways in trypanosomes with a focus on VSG proteins, antigenic variation and its important role in immune evasion. They are joined by Borges et al. for a targeted appraisal of the GPI anchor as a “universal evolutionary building block” for a diverse set of surface molecules in trypanosomes. Finally, Balmer and Faso review and place the limited data on unconventional protein secretion pathways involved in protist parasite virulence in a wider context.

Structures and processes at the host-parasite interface are notoriously difficult to analyze because the complex orchestration of interactions which have evolved with specific purposes requires investigating two very different organisms simultaneously. The growing community of scientists who explore molecular communications between pathogens and their hosts is addressing this challenge by driving the implementation of rapidly advancing technological developments. This allows researchers to study developmental processes and host-parasite interactions far beyond the mere uptake of nutrients and building blocks by parasites. Specifically, modern -omics approaches and cutting-edge microscopy have been very successful in bringing the term host-pathogen co-evolution to life on a molecular level, revealing the seemingly limitless ingenuity of both parasites and hosts in their perpetual arms race to evolve interaction strategies for colonizing or fending off invasion. However, omics-approaches depend heavily on the quality of parasite genome annotations which are wanting due to the large proportion of highly diverged genes and the richness of new inventions not present in well characterized model organism. The upshot is that a third or more returns from high-content -omics experiments with parasites remain non-interpretable which detracts significantly from the value of the investment in time and materials. Moreover, researchers tend to focus on the “known”, ignoring the big chunk of uncharted “dark data” in which the truly novel discoveries remain hidden, thus preventing a more complete understanding of structural and dynamic aspects of host-pathogen interactions. Therefore, despite producing comprehensive landscapes of gene and protein regulation which describing these interactions, the insights remain unsatisfyingly incomplete. Nevertheless, the power of genetic manipulation tools, especially CRISPR-Cas9 (when applicable), in combination with phenotype analysis, holds the promise of closing these gaps while assigning functions to hitherto uncharacterized parasite gene products. This may lead to the identification of novel therapeutic and diagnostic leads which would otherwise remain completely unknown.

